# Bottom-Up Control of Macrobenthic Communities in a Guanotrophic Coastal System

**DOI:** 10.1371/journal.pone.0117544

**Published:** 2015-02-13

**Authors:** Geraldina Signa, Antonio Mazzola, Valentina Costa, Salvatrice Vizzini

**Affiliations:** Department of Earth and Marine Sciences, University of Palermo, CoNISMa, via Archirafi 18, Palermo, Italy; Dauphin Island Sea Lab, UNITED STATES

## Abstract

Soft bottom macrobenthic communities were studied seasonally in three coastal ponds (Marinello ponds, Italy) at increasing distances from a gull (*Larus michahellis*) colony to investigate the effect of seabird-induced eutrophication (i.e. guanotrophication) on macrobenthic fauna. We hypothesized that enhanced nutrient concentration and organic load caused by guano input significantly alter the trophic and sedimentological condition of ponds, affecting benthic fauna through a bottom-up control. The influence of a set of environmental features on macrobenthic assemblages was also tested. Overall, the lowest macrobenthic abundances and functional group diversity were found in deeper sites, especially in the pond characterised by severe guanotrophication, where the higher disturbance resulted in a decline in suspension feeders and carnivores in favour of deposit feeders. An increase in opportunistic/tolerant taxa (e.g. chironomid larvae and paraonids) and totally azoic sediments were also found as an effect of the harshest environmental conditions, resulting in a very poor ecological status. We conclude that macrobenthic assemblages of the Marinello coastal system display high spatial variability due to a synergistic effect of trophic status and the geomorphological features of the ponds. The macrobenthic response to guanotrophication, which was a clear decrease in abundance, diversity and trophic functional groups, was associated with the typical response to severe eutrophication, magnified by the geomorphological features.

## Introduction

Fluxes of nutrients are of paramount importance in ecological systems due to the implications for primary production, trophic structure and biodiversity [1]. Through their activities and movements, seabirds, which commonly use coastal ecosystems as nesting sites or corridors, release a large mass of nutrients and contaminants contained mainly in guano [2–5] and consequently may directly and/or indirectly affect the functionality of these systems. Indeed, an increase in plant abundance and biomass and a decrease in plant species richness have been observed in many seabird-inhabited terrestrial sites around the world [6, 7]. Despite the marked effect that seabirds would be expected to also have on aquatic ecosystems, most ecological studies on coastal avifauna have until now not been strongly ecosystem-oriented.

Avian-induced nutrient enrichment has been shown to enhance phytoplankton, periphyton and seagrass production in aquatic systems adjacent to bird colonies [4, 8, 9]. Consequently, in case seabirds are not directly involved in the local food webs as top predators, they may also have far-reaching effects on macrobenthic communities by bottom-up forces. Indeed, bird-induced bottom-up effects on primary producers may flow upward in trophic webs, affecting abundance and/or biomass of organisms at different trophic levels, as observed for marine zooplankton [10], polychaetes [11], nematods [12], chironomids [13], isopods [14] and fish [8]. Moreover, where bird abundance is very high [14, 15] and/or the systems are small and with scarce water renewal, such as coastal ponds and lagoons [4], the avian-induced nutrient enrichment can be magnified leading undesirable effects on the whole ecosystem, similarly to severe eutrophication, here-hence the term guanotrophication [16].

On the other hand, coastal ponds and lagoons feature intrinsic geomorphological, hydrological and sedimentological characteristics, which also play an important, and often synergistic, role in influencing biotic communities and the whole ecosystem functioning [17, 18]. Shallow depth, small tidal range, and high residence time favour a set of trophic and sedimentary conditions (i.e. high nutrient load and organic matter and mud accumulation) whose surplus, in turn, is among the main causes of oxygen depletion, redox potential drop and toxic by-product build-up at the water-sediment interface [19, 20]. All these conditions have been shown to lead to the decline of ecosystem environmental quality, which is reflected in the impoverishment of communities. Among all, soft-bottom macrozoobenthic communities respond rapidly to the above-mentioned stressors by decreasing faunal richness, abundance, and biomass [21–23], and changing species and trophic group distribution [19].

The coastal system of Marinello, situated along the north-eastern coast of Sicily (Italy, Tyrrhenian Sea, Mediterranean), is made of five small ponds, characterised by a dynamic shape and size, due to rapid evolution of the coastal morphology. The innermost ponds are mainly influenced by surface run-off from surrounding lands and the outermost ones by indirect seawater inflows through infiltration or high waves during storms [24]. As for biotic input, the system is principally affected by the presence of a small resident colony of yellow-legged gull (*Larus michahellis*) that features a lower density in winter and spring (~85 ind. [4]) and a higher one in summer and autumn (~115 ind. [4]). For this reason, the Marinello system may be considered as a field laboratory for studying the effects of guanotrophication on soft bottom communities due to the different amount of subsidies the adjoining ponds receive from seabird guano. Through the analysis of isotopic tracers along the gradient of avian input, considerable seabird-derived nutrient load in the water column and sediment of the pond closest to the gull colony was detected [4]. Although the small size of the colony, the constant input of guano into this pond and its restricted nature definitely contributed to magnifying the effects expected in a naturally-stressed system [18]; triggering chronic guanotrophication [4], trace element contamination [25] and consequent trophic transfer to the biota [5]. No other bird colonies or migrating animals, which may represent other allochthonous input, have been recorded in proximity to the Marinello ponds. Among benthic fauna, only molluscs were previously studied [26], but results were not related to the gull colony input. Further, the whole macrobenthic community has never been investigated.

In an effort to fill some of these gaps, the main aim of this paper was to assess the effects of gull guano on soft-bottom macrobenthic communities in the Marinello coastal system. The hypothesis is that guanotrophication affects macrobenthic communities through bottom-up forces. Thus, the implications of these forces may materialise in different taxa dominance, abundance, diversity and trophic mode, to the point of influencing the environmental quality of the whole ecosystem. We tested this hypothesis by monitoring macrobenthic communities with a dual approach (taxonomic and functional), on a spatial and temporal scale in three ponds of the Marinello coastal system affected by different avian impact intensity. Furthermore, we assessed the relationships between the environmental characteristics (trophic, sedimentological, physico-chemical and geomorphological) of the ponds and the macrobenthic communities.

## Materials and Methods

### Ethics Statement

Samplings were conducted with permits from the Authority of the ‘‘Laghetti di Marinello’’ Nature Reserve (permit # 28599). No other permits were needed.

### Study area

**Figure 1:**
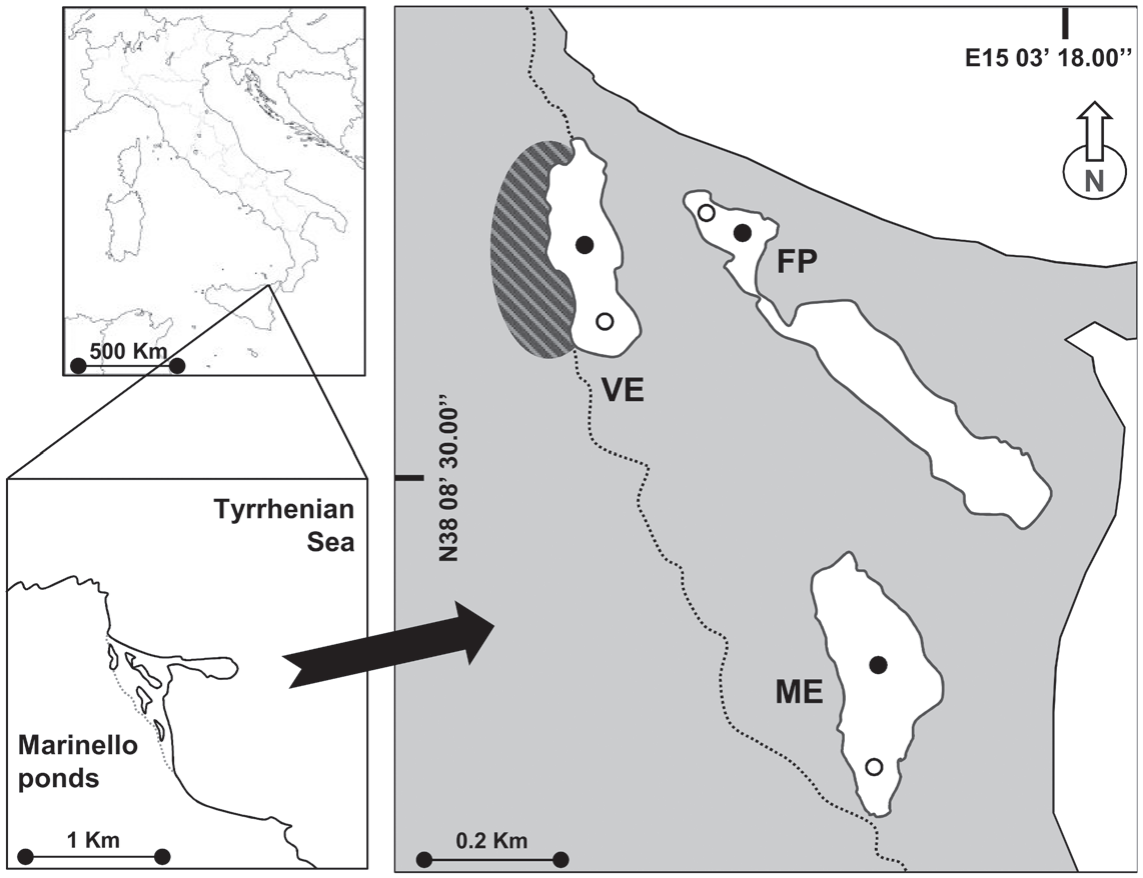
The study area of the Marinello ponds. The ponds studied were Verde (VE), Fondo Porto (FP) and Mergolo (ME) at increasing distance from the gull colony indicated by the striped oval. Sampling sites are also indicated: shore (white circle), bottom (black circle).

The Marinello coastal system is part of a Nature Reserve and a Site of Community Importance (code ITA030012) on the north-eastern coast of Sicily (Italy, Mediterranean Sea; Fig. 1). The system is made up of five small microtidal shallow brackish ponds (surface area from 1.3 to 4 ha, max depth from 2 to 4 m) (Verde, Fondo Porto, Porto Vecchio, Mergolo and Marinello), sparsely surrounded by reeds, separated from the sea by sandbars and lacking any direct freshwater and seawater input [24]. The overlying promontory of Tindari hosts one of the oldest resident colonies of yellow-legged gull (*Larus michahellis*) in Sicily. Although the ponds are adjoining, they differ greatly in the amount of subsidies they receive from seabird guano and one of the five ponds in particular, Verde, being adjacent to the colony, is affected by constant guanotrophication [4]. Three ponds were sampled in this study: Verde (hereafter VE) (N38 08.647; E15 02.882), Fondo Porto (hereafter FP) (N38 08.640; E15 03.022) and Mergolo (hereafter ME) (N38 08.362; E15 03.146), selected according to their distance from the gull colony. VE is the guanotrophic pond, situated just beside the gull colony, FP is about 200 m from the colony and gulls have been seen to walk around it, while ME is about 600 m from the colony and gulls have never been seen around it. The three selected ponds differ also for the distance from the sea, being FP nearer to the coastline, and VE and ME more landward (Fig. 1).

### Sampling and laboratory activities

Seasonally, from autumn 2008 to summer 2009, two sampling sites per pond, characterised by different ecological conditions, were chosen in accordance with previous studies [4, 5, 25], one situated along the shore (depth ≈ 0.5 m), the other at the bottom (depth: 2.0–3.0 m).

Sediment samples for macrobenthos analysis were collected using a Van Veen grab (Volume: 1.5 L; Surface: 0.03 m^2^; penetration depth: 10 cm), and samples for bulk sediment analysis were collected using hand corers (length: 25 cm; inner diameter: 4 cm). With both sampling devices, we randomly collected two replicates per site, and each replicate was made of 4 sediment samples. Before mixing the sediment to form the composite replicates, samples for macrobenthos were sieved through a 0.5 mm mesh. The first 10 cm of sediment corers were taken for grain size analysis, while pigments were analysed only in the surficial layer (0–1 cm). Depth and physico-chemical variables of bottom water (temperature, salinity, dissolved oxygen) were measured at each site using a multiparametric probe (Hydrolab Datasonde 5) and Eh at the water/sediment interface with a B&C Electronics 152.2 ORP portable meter. All samples were kept cool and dark upon arrival at the laboratory.

Once in the laboratory, macrobenthos samples were briefly placed in a narcotic/relaxant solution (7% magnesium chloride) and then fixed with a 4% buffered formaldehyde solution stained with rose Bengal, to facilitate macrobenthos identification. After one week, they were rapidly rinsed with sea water and stored in a 70% ethylic alcohol and 5% glycerine solution. Macrofaunal organisms were sorted, identified to the lowest possible taxonomic level (family, genus or species) under a stereomicroscope (10 to 40×), counted and preserved in 70% ethanol. Data were expressed as density (ind m^-2^). Chlorophyll pigments from bulk sediments were extracted in the dark following the same laboratory procedure used by Signa et al. [4] and sediment phaeopigments were determined according to Lorenzen [27]. Sediment grain size was determined following the same laboratory procedure described by Signa et al. [5].

### Data analyses

Among the 38 taxa identified, only 11 reported a relative abundance higher than 1% and accounted for 94% of the total abundance as a whole. For this reason, statistical analyses were carried out using only these 11 dominant taxa. A 3-way ANOVA (STATISTICA 10 package) was performed to test for spatial and seasonal differences in density of dominant taxa by setting pond, site and season as fixed factors. Sabellidae were excluded because these were found only in ME shore. Homogeneity of variances was previously checked using Cochran’s C-test and, when necessary, data were log(x+1) transformed to reduce heterogeneity to acceptable levels. When significant differences were found, post-hoc multiple comparisons were performed using the Fisher’s LSD test (least significant differences). Cluster analysis (group average linkage method) was carried out on Bray Curtis similarities calculated from the fourth-root-transformed density of the 11 dominant taxa using the PRIMER v6 package [28] to display similarities of macrobenthos density between ponds, sites and seasons. Azoic samples were excluded from the analysis. The similarity profile (SIMPROF) permutation test was incorporated into the cluster analysis to evaluate significant differences between groups. A similarity percentage test (SIMPER) was conducted to identify the taxa that contributed most to differences within and among groups resulting from cluster analysis. Differences between groups were tested for significance using analysis of similarity (ANOSIM).

To assess if guanotrophication affects the benthic communities in terms of trophic group distribution, all macrobenthic taxa identified were grouped into the following feeding modes: filter/suspension feeders, detritivores, deposit-feeders, grazers, carnivores and omnivores, based on a literature survey. When literature data were not available, information on feeding modes and behaviour was taken from the online World Register of Marine Species (www.marinespecies.org). Variability of trophic group distribution was assessed on a spatial and temporal basis.

BITS, the Benthic Index based on Taxonomic Sufficiency [29], seemed particularly suitable for assessing the ecological quality status (EcoQ) of the Marinello ponds, as it was specifically developed for microtidal and non-tidal lagoon systems, according to the tolerant/opportunistic approach. Indeed, BITS is given by the sum of two terms which account for (a) the distribution of the F families in three adaptability/tolerance classes (*nI, nII* and *nIII*) and (b) the distribution of the N specimens in the three classes of families (*fI, fII* and *fIII*). As such, BITS has the important advantage of taking into account both functional and quantitative aspects of the whole macrobenthic community. Further, EcoQ classes (High, Good, Moderate, Poor, Bad; *sensu* EU Water Framework Directive, WFD 2000/60/EC) were defined based on the BITS index achieved and the sediment type (i.e. muddy or sandy). In this study, BITS was calculated separately for each of the four seasons, three ponds and two sites of Marinello ponds, by pooling together the two replicates obtained in each site. Five families (Aclididae, Chitonidae, Holothuriidae, Littorinidae and Solemyidae) not included in the BITS master list were not taken into account in the computation of the index. We presumed that their low abundances (< 0.2%) did not affect the results.

To assess the influence of a set of “best matching” environmental variables that explain the community pattern in a multivariate context [28], the Spearman rank correlation coefficient was evaluated using the BIO-ENV routine. This method is based on rank correlations between Euclidean distance similarity matrices of environmental variables and Bray-Curtis similarity matrices from macrobenthic abundance data, and reduces the number of variables related to the observed benthic pattern, highlighting those that play the most important role. The environmental variables included in the analysis were: trophic (chlorophyll pigments, total nitrogen and phosphorus), sedimentological (grain size, total organic carbon, hereafter TOC, and trace element concentrations), physico-chemical (temperature, salinity, dissolved oxygen, Eh) and geomorphological (surface area and depth). Nitrogen and phosphorus data, which have been found to be linked to guanotrophication, were taken from Signa et al. [4] and TOC and trace element (i.e. As, Cd, Cr, Cu, Ni, Pb, THg, V, Zn) data from Signa et al. [25]. Subsequently, the relationships between macrobenthic communities and TOC, one of the most important environmental stressor for benthic fauna in coastal areas [22, 30, 31] were assessed through correlations between TOC and three benthic response indicators: total density N, Shannon’s diversity index H’ and the benthic index BITS.

## Results

### Spatio-temporal variability of macrobenthic communities

**Figure 2:**
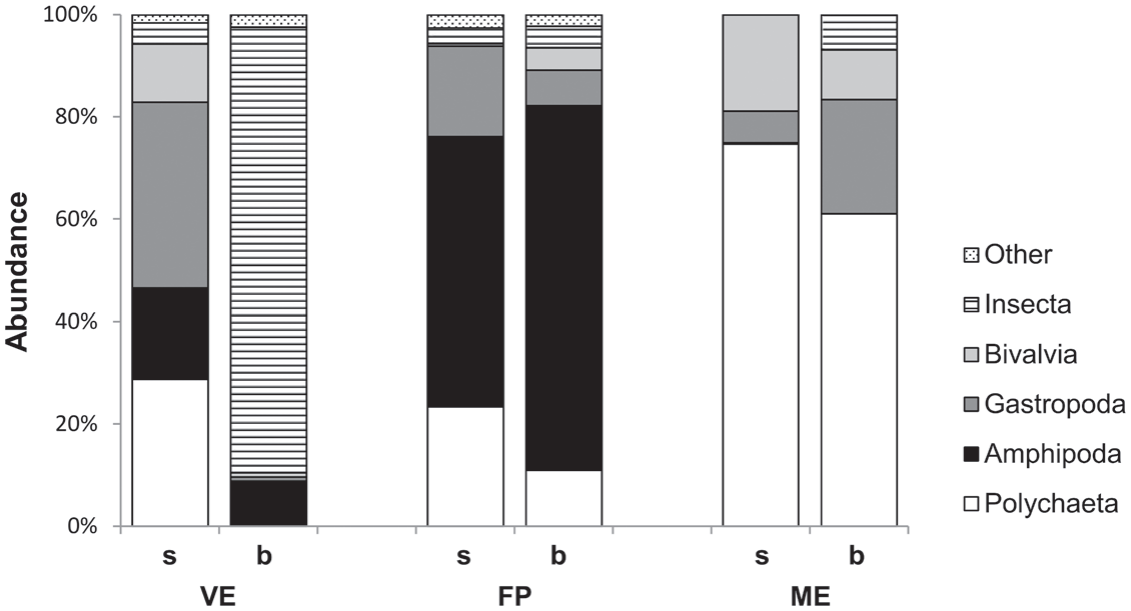
Abundance (%) standardised to 100% of macrobenthic taxa in the shore (s) and bottom (b) sites of the Marinello ponds: Verde (VE), Fondo Porto (FP) and Mergolo (ME).

**Figure 3:**
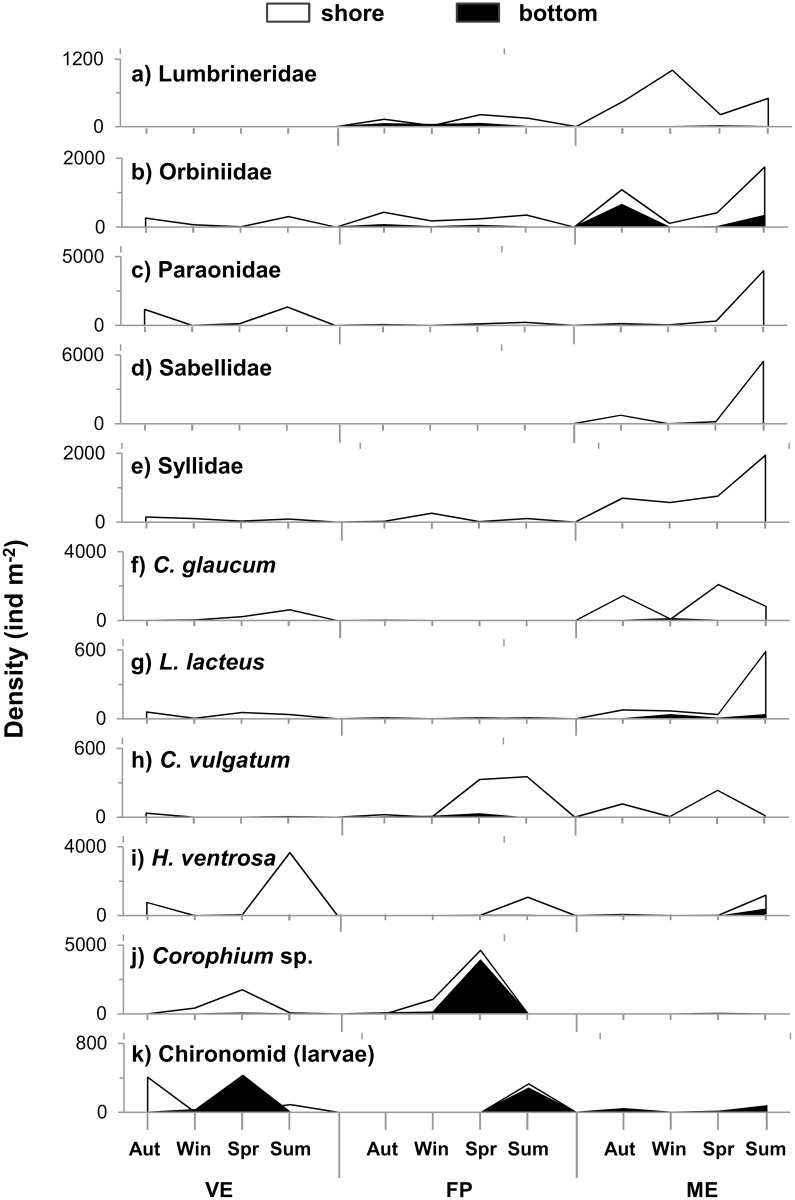
Density (individual m^-2^) of the most abundant taxa (>1%; 94% of total abundance) sampled throughout the sampling seasons in the Marinello ponds: Verde (VE), Fondo Porto (FP) and Mergolo (ME). Different colours indicate the sampling sites: shore (white area), bottom (black area).

A total of 120 952 individuals from 38 taxa were collected in the Marinello ponds throughout the study period. The guanotrophic pond, VE, showed a quite diverse taxa composition in the shore site and a marked dominance of chironomid larvae in the bottom site (the bottom patterns refers exclusively to winter and spring because azoic conditions were found in autumn and summer). In contrast FP, the adjacent pond, was dominated by amphipods and ME, the pond furthest from the gull colony, was dominated by polychaetes with similar patterns in both sites (Fig. 2). The most abundant taxa found in the whole system were the polychaete families Lumbrineridae, Orbiniidae, Paraonidae, Sabellidae and Syllidae, the bivalves *Cerastoderma glaucum* and *Loripes lacteus*, the gastropods *Cerithium vulgatum* and *Hydrobia ventrosa*, the amphipod *Corophium* sp. and the chironomid insect larvae. Density of the dominant taxa varied widely across the sites, ponds and seasons (Fig. 3, Table 1). All the dominant taxa, except chironomid larvae, were significantly more abundant in the shore sites than in the bottom ones. The most abundant taxa in the guanotrophic pond, were the small gastropod *H. ventrosa*, which showed a marked, although not significant, peak at the shore site, during summer (Fig. 3 i) and chironomid larvae with the highest densities in both sites (Fig. 3 k). The amphipod *Corophium* sp. and the polychaete family Paraonidae were also well represented in VE, although the highest abundances were recorded in FP and ME respectively (Fig. 3 j, c). FP, the adjacent pond, was also characterised by the highest abundance of *C. vulgatum* (Fig. 3 h) while ME, the pond far from the gull colony, by the highest abundance of all the polychaetes families (Fig. 3 a-e) and both bivalves (Fig. 3 f-g), reaching the highest densities in both spring and summer.

**Table 1:** ANOVA of the dominant taxa abundance.

**Source of variation**	**Df**	**Lumbrineridae**			**Orbiniidae**			**Paraonidae**			**Syllidae**			***C. glaucum***		
		**MS**	**F**	**p**	**MS**	**F**	**p**	**MS**	**F**	**p**	**MS**	**F**	**p**	**MS**	**F**	**p**
season	3	1.95	1.60	n.s.	10.26	8.63	***	20.29	6.61	**	0.52	0.25	n.s.	1.75	0.38	n.s.
Pond	2	55.35	45.45	***	38.37	32.26	***	7.90	2.58	n.s.	15.23	7.14	**	24.99	5.46	*
Site	1	69.07	56.72	***	113.75	95.61	***	144.80	47.18	***	208.05	97.55	***	47.20	10.31	**
season*pond	6	1.31	1.08	n.s.	7.49	6.30	***	4.66	1.52	n.s.	1.16	0.55	n.s.	3.60	0.79	n.s.
season*site	3	2.75	2.25	n.s.	4.07	3.42	*	12.33	4.02	*	4.52	2.12	n.s.	4.82	1.05	n.s.
pond*site	2	33.38	27.41	***	1.81	1.52	n.s.	2.81	0.91	n.s.	17.86	8.38	**	26.24	5.73	**
season*pond*site	6	2.74	2.25	n.s.	2.67	2.25	n.s.	3.52	1.15	n.s.	0.76	0.36	n.s.	4.51	0.99	n.s.
Total	47															

Source of variation	df	*L. lacteus*			*C. vulgatum*			*H. ventrosa*			*Corophium* sp.			Chironomidae (larvae)		
		MS	F	p	MS	F	p	MS	F	p	MS	F	p	MS	F	p
season	3	25177.20	16.50	***	5.28	1.75	n.s.	38.17	9.42	***	45.11	19.16	***	31463.62	0.93	n.s.
Pond	2	48013.68	31.46	***	13.77	4.55	*	1.42	0.35	n.s.	57.63	24.48	***	45446.95	1.35	n.s.
Site	1	62076.31	40.68	***	20.46	6.76	*	33.88	8.36	**	15.32	6.51	*	24.08	0.00	n.s.
season*pond	6	25441.95	16.67	***	2.70	0.89	n.s.	2.37	0.58	n.s.	10.20	4.33	**	55248.24	1.64	n.s.
season*site	3	22449.41	14.71	***	7.11	2.35	n.s.	14.10	3.48	*	7.85	3.33	*	34687.81	1.03	n.s.
pond*site	2	32838.05	21.52	***	3.30	1.09	n.s.	10.57	2.61	n.s.	7.72	3.28	n.s.	2993.23	0.09	n.s.
season*pond*site	6	21144.33	13.86	***	2.54	0.84	n.s.	2.48	0.61	n.s.	3.19	1.36	n.s.	39825.92	1.18	n.s.
Total	47															

All data, except *L. lacteus* and chironomids, were log (x+1) transformed. Sabellidae were excluded from the analysis because found only in ME shore.

*: p<0.05;

**: p≤0.01;

***: p<0.001,

n.s.: not significant.

**Figure 4:**
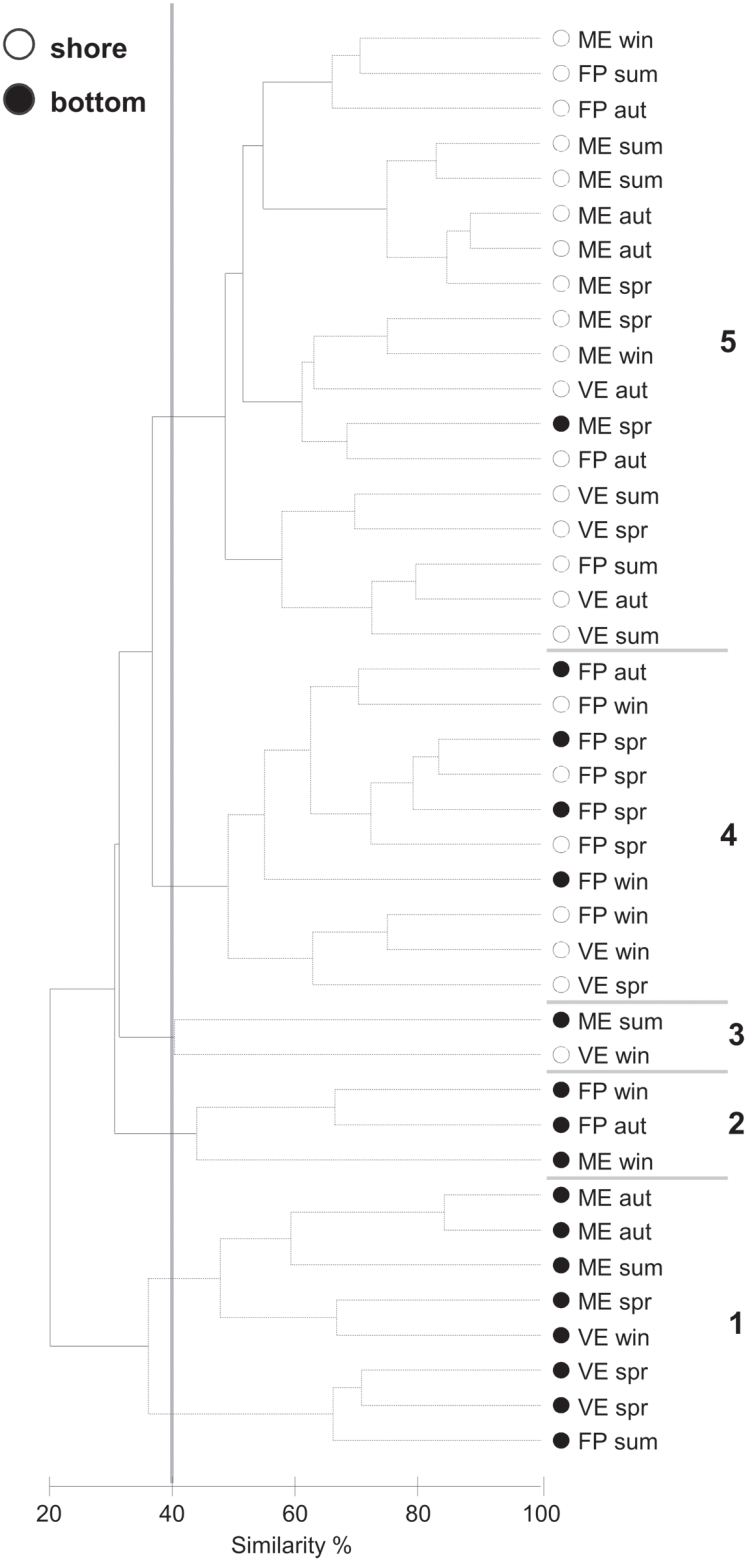
Dendrogram showing the hierarchical clustering (group average linking) of the macrobenthic fauna in the Marinello ponds throughout the sampling seasons. 7 samples (VE aut R1, 2; VE win R2; VE sum R1, 2; FP sum R1; ME win R1; all from bottom sites) were excluded from the analysis as they were azoic. The Bray-Curtis similarity index was calculated from the fourth root-transformed abundances of the 11 most dominant taxa. Black branches indicate significant groups according to the SIMPROF test; dotted lines indicate non-significant groups. 5 significant groups (1–5) were identified below the similarity level of 40%. Different colours of the symbols indicate the sampling sites: shore (white circle), bottom (black circle).

On the cluster dendrogram, five clusters (sample groups 1–5) were defined at 40% similarity level (Fig. 4). One-way ANOSIM detected significant differences in community structure (global R = 0.778, p<0.001) and between all pairs of groups (p<0.05) except one (groups 2 and 3: p = 0.1). Almost all the samples from bottom sites grouped together (clusters 1 and 2). VE and ME bottom featured the cluster 1 where chironomid larvae was the most typical taxon, contributing 76.7% to the within-group similarity, as revealed by the SIMPER procedure. Cluster 2 consisted essentially of FP and ME bottom samples and the molluscs *C. glaucum* and *C. vulgatum* contributed 46.0 and 38.7% respectively to the within-group similarity. Cluster 3 grouped the samples based on orbinid abundance, the contribution of which to the within-group similarity was total (100%). Cluster 4 contained samples from both sites of FP and a few from the shore of VE, where the amphipod *Corophium* sp. contributed most (55.9%) to the within-group similarity. Cluster 5 was characterised almost exclusively by samples from ME shore, mixed with FP and VE shore, that grouped together based on the abundance of polychaete families (Orbiniidae, Syllidae, Paraonidae and Lumbrineridae), contributing to the within-group similarity 70.3% as a whole.

**Figure 5:**
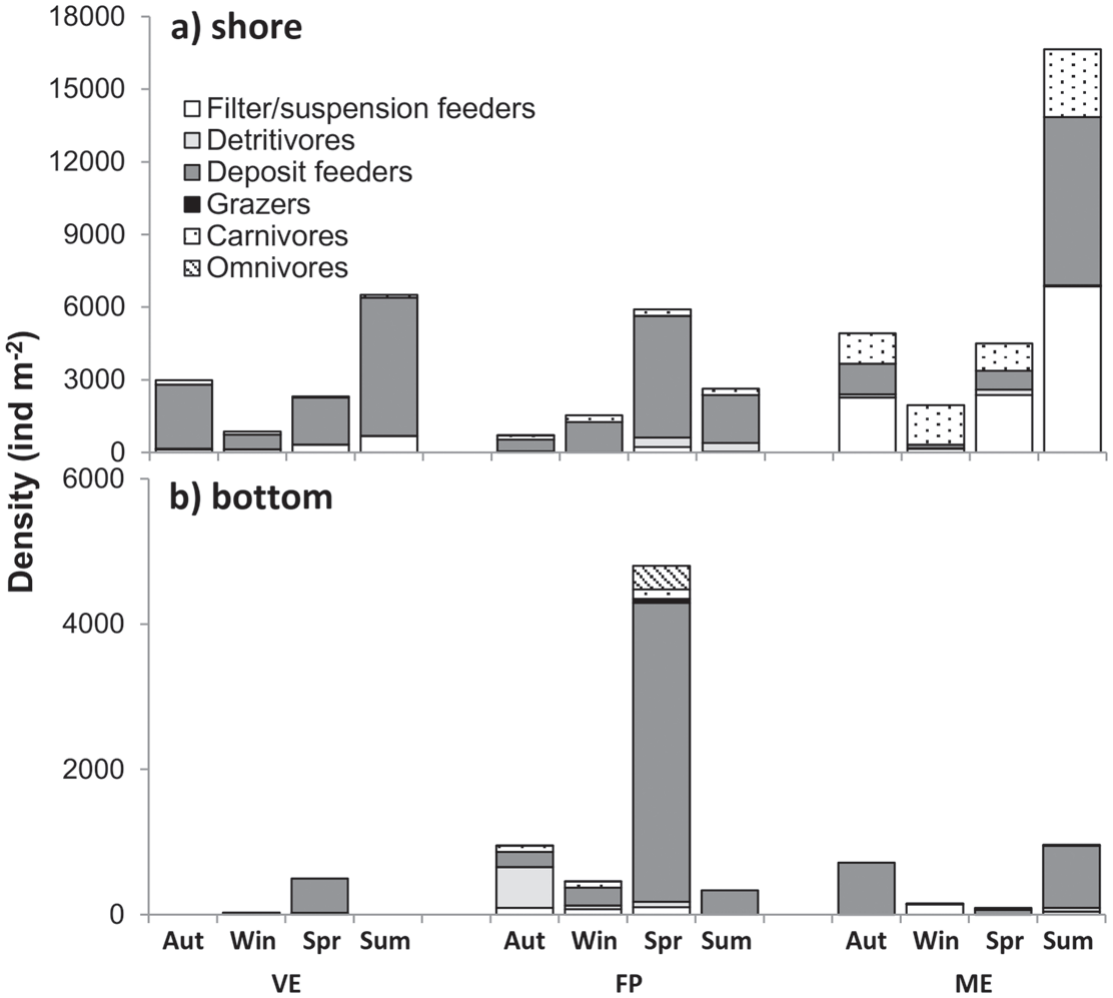
Density (individual m^-2^) of macrobenthic fauna grouped by feeding mode throughout the sampling seasons in the Marinello ponds: Verde (VE), Fondo Porto (FP) and Mergolo (ME).

Benthic taxa grouped by feeding mode exhibited different spatial and temporal patterns (Fig. 5). In the shore sites, deposit feeders exhibited a clear dominance over the other groups in the ponds closest to the gull colony, VE and FP, while carnivores, deposit feeders and filter/suspension feeders were almost equally abundant in the other pond, ME. In the bottom sites, feeding mode diversity was reduced in all ponds exhibiting almost exclusively deposit feeders, although FP showed higher diversity especially in spring. Overall, in both sites of all ponds, the seasonal trend in total faunal density reflected that of the numerically dominant trophic group and differed between FP and the other two ponds. A peak in density was shown in spring at FP and in summer at both VE and ME.

### H’ and BITS indices and ecological quality status (EcoQ)

In the bottom site of the guanotrophic pond VE, H’ and BITS were very low in spring and even null in autumn, winter and summer because sediment was azoic or almost (Table 2). Overall, the lowest H’ values (≤ 0.5) were associated with the bottom sites, in VE in particular, throughout the whole sampling period, while in the non-guanotrophic ponds, FP and ME, only in summer and in autumn/winter respectively. In contrast, the highest values (>1.5) were recorded in ME shore and FP bottom (autumn/winter). The trend in BITS was similar to that in H’, and consequently the worst ecological quality status (i.e. Poor/Bad) was recorded in VE bottom in all the seasons and sporadically in FP and ME bottom. In contrast, the best EcoQ status (i.e. Good/High) was achieved most frequently in both sites of the non-guanotrophic ponds, and only sporadically in VE shore.

**Table 2:** Benthic index BITS [29], EcoQ classes (H: High, G: Good; M: Moderate; P: Poor; B: Bad) and Shannon’s diversity index calculated separately for each of the seasons, sites and Marinello ponds: Verde (VE), Fondo Porto (FP) and Mergolo (ME).

***Pond***	***Site***	***Season***	***Sediment type***	***Abundance N (ind m^-2^)***	***fI***	***fII***	***fIII***	***nI***	***nII***	***nIII***	***BITS***	***EcoQ***	**H′ (log_e_)**
VE	shore	aut	S	5969.4	11.1	36.3	52.6	6	3	2	1.2	M	1.2
		win	S	1717.7	23.3	76.7	0.0	3	5	0	3.2	H	1.2
		spr	S	4625.9	3.7	89.9	6.4	5	5	2	1.8	G	0.6
		sum	S	13018.7	2.0	76.2	21.8	5	6	2	1.3	M	1.5
	bottom	aut	M	0.0	0.0	0.0	0.0	0	0	0	-	B	0.0
		win	M	59.5	0.0	0.0	100.0	0	0	1	0.0	B	0.0
		spr	M	994.9	4.3	9.4	86.3	3	2	1	0.7	P	0.5
		sum	M	0.0	0.0	0.0	0.0	0	0	0	-	B	0.0
FP	shore	aut	S	1411.6	9.0	83.1	7.8	4	4	1	1.8	G	1.1
		win	S	3069.7	18.3	81.7	0.0	2	3	0	3.0	H	0.8
		spr	S	11828.2	12.5	85.8	1.7	9	4	1	2.7	H	0.9
		sum	S	5263.6	18.9	60.1	21.0	6	6	2	1.6	G	1.5
	bottom	aut	S	1904.8	72.3	25.0	2.7	9	7	2	2.9	H	2.1
		win	S	909.9	50.5	49.5	0.0	6	6	0	3.5	H	1.8
		spr	S	9591.8	8.2	91.7	0.2	13	8	2	3.0	H	0.9
		sum	S	663.3	0.0	15.4	84.6	0	2	1	0.2	B	0.2
ME	shore	aut	S	9829.9	33.3	63.6	3.1	8	6	2	2.6	H	1.8
		win	S	3911.6	31.1	64.8	4.1	5	4	3	2.3	H	1.3
		spr	S	9005.1	29.2	62.3	8.5	9	6	2	2.2	H	1.7
		sum	S	33299.3	45.8	29.2	25.0	9	6	3	1.8	G	1.7
	bottom	aut	S	1428.6	0.6	93.5	6.0	1	2	1	1.5	M	0.3
		win	S	306.1	8.3	91.7	0.0	3	2	0	2.9	H	0.4
		spr	S	187.1	18.2	31.8	50.0	1	3	2	0.9	P	1.0
		sum	S	1921.8	6.6	82.3	11.1	2	4	2	1.5	M	1.0

Sediment type (S: sandy; M: muddy) is indicated. Total abundance N (ind m^-2^) and benthic parameters needed to calculate BITS index are also indicated: the frequency (%) of the specimens belonging to sensitive, tolerant and opportunistic families (fI, fII and fIII); the number of sensitive, tolerant and opportunistic families in the sample (nI, nII and nIII).

### Relationship between environmental variables and macrobenthic communities

Table 3 shows the average environmental variables (geomorphological, physico-chemical, trophic and sedimentological) detected in the Marinello ponds throughout the study period. Salinity decreased from FP to VE to ME, reflecting the increasing distance from the sea. The highest chlorophyll-*a* and phaeopigment concentration values were recorded in VE bottom, due to the high trophic status and primary production, which are symptoms of guanotrophication [4]. Sediment texture was largely sandy in the whole area, except in VE and ME bottom, where a high mud content was found. In these same sites, the lowest dissolved oxygen and redox potential were also recorded. The BIO-ENV analysis, which also included variables from previous studies (cfr. materials and methods section), revealed that both trophic variables (phaeopigment and nitrogen concentration) and geomorphological/sedimentary ones (depth and gravel content) were the combination of environmental variables which produced the strongest Spearman correlation with the abundances of benthic taxa (r = 0.606). Dissolved oxygen, mud and TOC concentration also showed similar correlations (r = 0.600; 0.600; 0.596 respectively), while among trace elements, only Pb concentrations correlated with macrobenthic community (r = 0.600).

**Table 3:** Geomorphological, physico-chemical, trophic and sedimentological variables (mean ± s.d.) in the different sites of the Marinello ponds throughout the study period.

**Pond**	**VE**			**FP**			**ME**		
**Site**	**shore**		**bottom**	**shore**		**bottom**	**shore**		**bottom**
Surface area (Ha)		1.7			1.3			2.5	
Depth (m)	0.5 ± 0.1		2.5 ± 0.2	0.5 ± 0.1		2.0 ± 0.3	0.5 ± 0.1		3.0 ± 0.4
Temperature (°C)	23.5 ± 6.1		23.2 ± 5.4	24.6 ± 5.5		24.2 ± 5.4	23.3 ± 6.4		24.0 ± 4.5
Salinity (ppt)	34.0 ± 2.1		35.1 ± 2.1	36.7 ± 1.3		36.7 ± 1.3	31.7 ± 4.8		33.0 ± 2.4
LDO% (Sat)	100.4 ± 15.5		84.2 ± 21.2	99.1 ± 38.1		96.5 ± 29.9	105.7 ± 14.0		84.5 ± 46.2
Eh (mV)	140.0 ± 52.7		-159.0 ± 96.8	121.6 ± 36.4		-92.6 ± 163.6	66.9 ± 120.6		-185.1 ± 107.7
Chlorophyll-*a* (μg g^-1^)	2.1 ± 1.8		11.3 ± 3.7	2.1 ± 0.7		2.5 ± 1.7	10.3 ± 5.8		2.1 ± 1.3
Phaeopigments (μg g^-1^)	1.4 ± 1.5		61.3 ± 14.9	3.7 ± 3.0		2.9 ± 2.1	10.1 ± 4.2		20.8 ± 4.0
Mud (%)	0.4 ± 0.1		48.6 ± 9.6	8.5 ± 6.5		1.3 ± 0.4	13.5 ± 1.1		33.8 ± 1.7
Sand (%)	96.5 ± 1.5		33.1 ± 1.1	85.6 ± 10.8		97.5 ± 0.0	73.3 ± 2.3		56.0 ± 0.9
Gravel (%)	3.1 ± 1.4		18.3 ± 10.7	6.0 ± 4.3		1.3 ± 0.4	13.3 ± 3.5		10.2 ± 2.6

**Figure 6:**
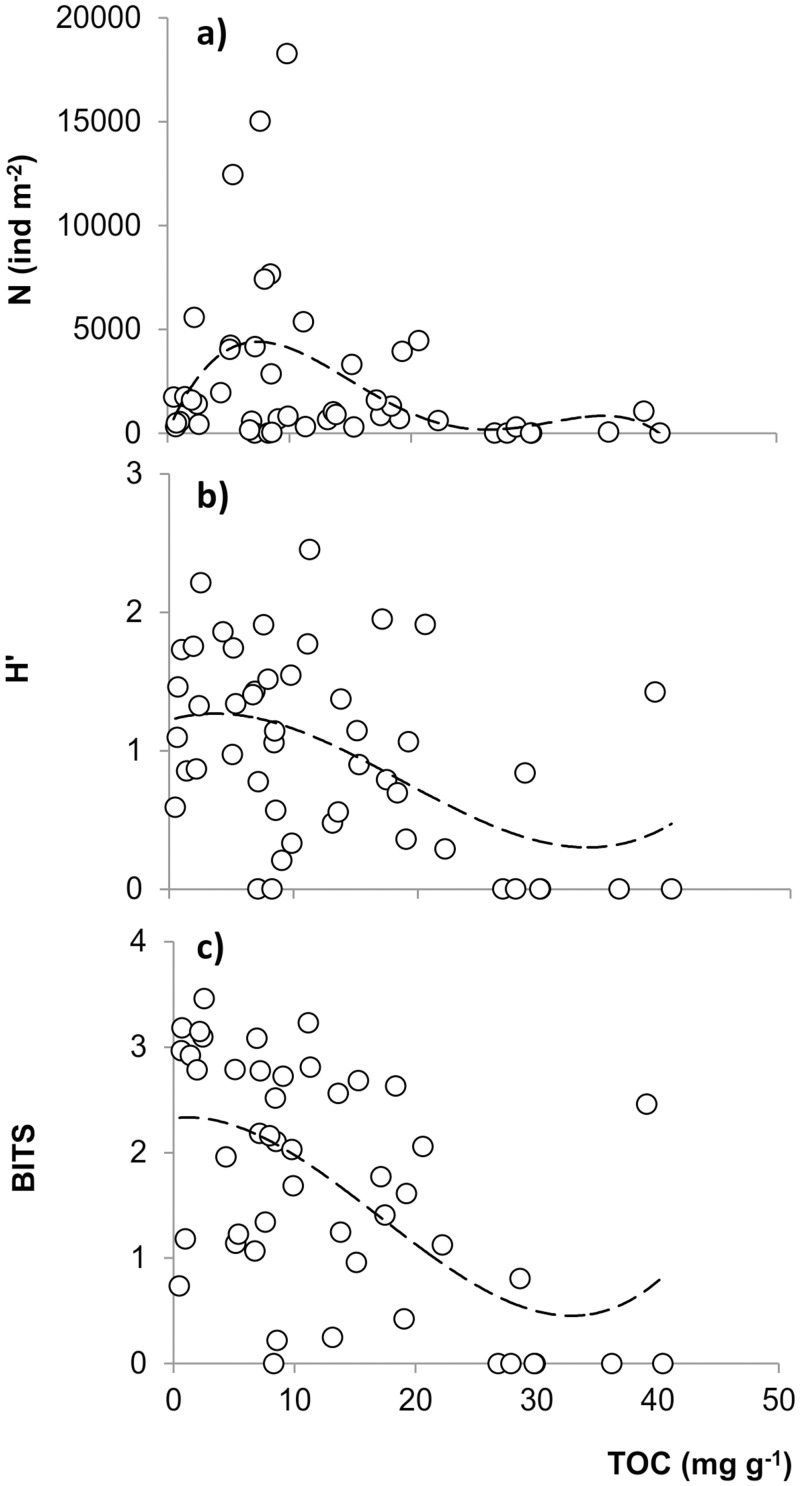
Distribution of various measures of benthic macrofaunal response along a TOC gradient: (a) Total abundance N; (b) Shannon diversity H’; (c) BITS. All correlations were described by polynomial functions. N-TOC: y = -0.0498x^4^ + 4.6137x^3^ - 139.65x^2^ + 1392.5x + 26.339 R = 0.41. H’-TOC: y = 7E-05x^3^ - 0.004x^2^ + 0.0269x + 1.2213; R = 0.47. BITS-TOC: y = 0.0001x^3^ - 0.006x^2^ + 0.0137x + 2.3257; R = 0.57.

The relationship between total macrobenthic abundance N and TOC was best described by a bimodal curve where the first and highest peak fell within the TOC concentration range: 5–10 mg g^-1^ and a second and smaller peak corresponded with the TOC concentration range: 35–45 mg g^-1^ (Fig. 6 a). The correlation was described by a polynomial function (y = -0.0498x^4^ + 4.6137x^3^ - 139.65x^2^ + 1392.5x + 26.339; R = 0.41). Slightly stronger correlations were found between both the Shannon index H’ and BITS and TOC concentration following a polynomial function (H’-TOC: y = 7E-05x^3^ - 0.004x^2^ + 0.0269x + 1.2213; R = 0.47; BITS-TOC: y = 0.0001x^3^ - 0.006x^2^ + 0.0137x + 2.3257; R = 0.57) (Fig. 6 b, c). Both correlations showed a similar pattern, with only one mode corresponding to TOC values of around 5 mg g^-1^, followed by a gradual decrease up to a final inflection point of between 30 and 40 mg g^-1^.

## Discussion

### Spatial and temporal variability in dominant taxa and feeding guilds

The soft bottom macrobenthic communities of the Marinello coastal system showed a marked inter-pond variability. In particular, VE, the guanotrophic pond [4], displayed distinctive features, compared with the other ponds, with high diversity and density of benthic taxa at low depth (i.e. shore) and a drastic reduction in both parameters up to azoic conditions at higher depth (i.e. bottom). Macrobenthic community in VE was dominated by a few stressor-tolerant and opportunistic taxa during the whole sampling period, giving indication of a long-term severe disturbance. Indeed, the two stressor-tolerant species *Corophium* sp. and *Hydrobia ventrosa* [29, 32], dominant at low depth in spring and summer respectively, are typical of highly disturbed and organic-enriched coastal environments [23, 33, 34]. Another dominant taxon along the shores of VE was the opportunistic polychaete paraonid [29, 32], which is reported to be responsive to phytodetritus pulse [35], condition reported in VE as an effect of the avian input [4]. Contrarily to the shores, the opportunistic chironomid larvae have been found almost exclusively at the bottom of VE. Tolerance to adverse conditions such as low levels of dissolved oxygen, high organic load, high silt content and sediment-bound toxin concentration, typical conditions of severely eutrophic and polluted areas, such as those described in the deeper part of VE [4], are reported to promote the outburst of chironomid larvae populations in aquatic ecosystems [36, 37]. Further, increase in chironomid larvae abundance has also been related to increased seabird-delivered nutrients in Arctic ponds and Baltic sea coastal waters [14, 38]. *Corophium* sp. and chironomid larvae were also abundant in the adjacent pond FP, respectively in spring and summer, together with the gastropod *Cerithium vulgatum.* Unlike the above-mentioned taxa, *C. vulgatum* is reported to be a sensitive species [29, 32], that lives in marine/estuarine habitats in association with seagrass and macroalgal beds [39, 40]. The presence of sensitive species and of dense macrophyte beds in the whole pond (pers. obs.) gives an overall indication of healthier benthic conditions and lower eutrophication levels. Polychaetes, usually very common in coastal lagoons [23, 41], were the most abundant taxonomic group in the pond far from the gull colony, ME. According to the tolerant/opportunistic approach, the most abundant families detected in ME, Sabellidae and Syllidae were sensitive, Lumbrineridae and Orbiniidae tolerant and only Paraonidae opportunistic [29], while the classic opportunistic polychaetes, able to proliferate after increases in organic matter (i.e. Capitellidae, Cirratulidae, and Spionidae) were scarce or absent, indicating a natural eutrophic, but not dystrophic, condition.

As regards temporal variation in benthic abundance, seasonal trends were different overall between FP and the other two ponds, VE and ME. Soft-bottom benthic assemblages usually undergo seasonal fluctuations that follow a general pattern of regression (summer/autumn) and subsequent recovery (winter/spring) [23, 41]. This is consistent with the pattern observed in the seaward FP, where spring peak was followed by a catastrophic summer decline, presumably due to the harsh conditions created by the decaying vegetal material associated with warmer temperatures [42, 43]. In the more eutrophic/guanotrophic and landward ponds, VE and ME, the decline was shifted to autumn/winter and the following recovery to spring/summer. Being these ponds phytoplankton dominated, it is not surprising that the increased and prolonged planktonic production may lead to a continual, positive effect on benthic secondary production. A close relationship in seasonally-driven trophodynamic processes among primary producers and benthic consumers was also observed by Magni et al. [43] in estuarine environments.

Observing the macrobenthic assemblages of the Marinello ponds from a functional standpoint, a decrease in carnivores and suspension/filter feeders, accompanied by an increase and marked dominance of deposit feeders was evident from the less seabird-impacted pond to the guanotrophic one. The massive avian-induced phytodetritus deposition in VE [4] and the high macrophyte abundance in the adjoining FP (pers. obs.) may explain the deposit feeder dominance in these two ponds. All the common and most abundant taxa are known to be opportunistic or unselective deposit feeders: chironomids feed mainly on diatoms and detritus [37], or directly on living, newly sedimented phytoplankton [38, 44]; *H. ventrosa* is an epibenthic browser on the top sediment layer [45, 46], while *Corophium* sp., orbinids and paraonids feed unselectively on the subsurface of sediment [35, 46, 47]. *C. volutator*, one of the most common co-genus species in Mediterranean lagoons has been described both as an unselective deposit-feeder and as a detritivore, using diatoms, macrophyte detritus [40, 48] and/or sediment [49] as a source of organic matter.

The observed variability of dominant taxa and trophic guilds in the Marinello ponds along the avian input recalls the classic theoretical models describing biological succession along environmental gradients [30, 50] and is consistent also with numerous applied studies and reviews on benthic communities associated with eutrophication [e.g. 51,52]. In eutrophic Greek lagoons, Reizopoulou and Nicolaidou [53] also noticed a shift in benthic biomass and size along a eutrophication gradient: from larger-bodied specimens (mostly filter-feeding bivalves and carnivorous polychaetes) to small-bodied specimens (mainly tolerant and opportunistic deposit feeders). Although we did not analyse macrobenthic biomass and size, our results may be consistent with those: the big-bodied filter-feeder bivalve *Cerastoderma glaucum* and the carnivorous polychaetes Lumbrineridae and Syllidae were abundant only in ME and decreased in the other ponds in favour of small-bodied deposit-feeders (i.e. *H. ventrosa*, *Corophium* sp., chironomid larvae).

### Environmental factors affecting soft bottom macrobenthic communities

BIO-ENV analysis revealed that both the trophic status and geomorphology of ponds play an important role in influencing soft bottom macrobenthic assemblages of the Marinello ponds. Among the trophic variables, nitrogen and phaeopigment concentrations were the most important environmental stressors in driving change in the macrobenthic communities and both are related to a high trophic status. Together with phosphorus, nitrogen is the main component responsible for eutrophication in coastal areas [54] and in particular for guanotrophication in VE [4] because of its high concentration in guano [11]. Phaeopigments are the early degradation product of chlorophyll pigments, and these usually peak during and following phytoplankton blooms. As phaeopigments degrade more slowly than chl-*a*, their outstanding concentration and the consequent low chl-*a*/phaeo ratio (0.2 ± 0.1) in the bottom sediment of the guanotrophic pond, confirm that large deposition of phytoplankton takes place and a large amount of phytoplankton-derived organic matter accumulates in sediments. This scenario is consistent with a marked influence from guanotrophication and the consequent phytoplankton bloom and phytodetritus deposition onto the sediment on macrobenthic communities, confirming the previous discussion on dominant taxa and feeding mode distribution.

Among the geomorphological variables, depth was the most important in structuring the soft bottom macrobenthos of the Marinello ponds. The effect of depth in reducing benthic abundances and impoverishing the whole assemblage was clear in both landward ponds, guanotrophic and not (VE and ME), which share the typical geomorphological structure of coastal ponds (i.e. conical shape). Depth was less important in FP because of its intrinsic homogeneity and shallowness. The deepest sites in coastal ponds with scarce water exchange are more subject to high sedimentation rate, organic matter and mud accumulation. Indeed, VE and ME bottom featured the highest mud content and the lowest dissolved oxygen percentage, variables that were also important in influencing the macrobenthic assemblages of the Marinello ponds. An excessive nutrient load and sediment organic matter surplus, the sedimentation rate of which exceeds the degradation rate, are among the main causes of oxygen depletion and build-up of toxic by-products (i.e. ammonia and dissolved sulphide) at the water-sediment interface of eutrophic coastal ponds [22, 30]. This is a crucial stage in the impoverishment of soft bottom macrobenthic communities, limiting faunal richness, abundance and biomass [20, 23, 33]. Thus, TOC is usually recognised among the main factors constraining composition, structure and distribution of soft bottom assemblages [22, 30, 31]. Indeed, in this study, TOC was among the variables influencing overall macrobenthic communities, and its relationship with macrobenthic abundance, diversity and the BITS index, (good indicators of benthic response to environmental stressors) was consistent with previous studies. The bimodal relationship found between macrobenthic abundance and TOC was in accordance with the pattern indicated by classic and recent studies [30, 31], with a primary peak of total abundance at low TOC concentrations and a secondary and lower peak at higher TOC concentrations. The secondary peak is consistent with the increase of fewer, but heartiest, opportunistic taxa and corresponds, indeed, to the major decline of macrobenthic diversity and BITS index. In addition to the above-mentioned sediment characteristics, Pb concentration also influenced the macrobenthic community structure of the Marinello ponds. Greater sediment concentrations of trace elements, which essentially stem from the guano input in the study area [5, 25], are reported to have deleterious effects on the biota through massive mortalities of benthic populations and altering the soft-bottom macrobenthic community structure [55, 56].

### Conceptual pattern of macrobenthic response in the Marinello ponds

In light of the previously discussed spatio-temporal variability of taxa/feeding guild and the influence of environmental factors highlighted in the Marinello ponds, the response of soft bottom macrobenthic communities to guanotrophication reminds the response to severe eutrophication. Indeed, the observed scenario may be assimilated into the classic conceptual model described by Pearson and Rosenberg [30], which is a decrease in abundance in line with trophic status followed by a peak in opportunistic taxa and a further decline up to a complete disappearance of benthic fauna. The overall outline of the situation of macrobenthic communities in the Marinello ponds was also well summarized by the BITS index which revealed the worst ecological status in the guanotrophic pond.

The clear effect of guano on the trophic processes previously found along the avian gradient of the Marinello ponds, that is a sharp decrease of trophic status and primary productivity at increasing distances from the gull colony [4], was not fully mirrored in the macrobenthic fauna because of the overlapping with geomorphological effects. Despite the intermediate position of FP along the avian gradient, it deviates from the other ponds because of the influence of several environmental features, such as smaller size, lower depth, higher seawater inflow and higher sand content [24], in influencing internal ecological processes. Thus, with reference to the response of coastal systems to increased eutrophication [54] and the theoretical models describing biological succession along environmental gradients [30, 50], the conceptual pattern of the ecological status and macrobenthic response in the Marinello ponds (taking into account only the akin ponds VE and ME) can be summarized as follows:

1) the not-guanotrophic ME represents the first/intermediate step in biological benthic succession, being a restricted system with scarce water renewal which shows the first signs of eutrophication: macrophyte reduction, TOC and mud increase and polychaete dominance. The EcoQ assessment and the high diversity of both taxa and feeding guilds suggest that ME is still a healthy coastal pond;
2) the guanotrophic VE represents the advanced/final step in biological succession as an effect of the increased seabird-mediated nutrient input. As nutrient load increases, phytoplankton biomass and trophic status increase and the large amount of phytodetritus produced in the system sinks to the seabed, affecting the sediment compartment and the resident biota. Sensitive and tolerant species abundance decreases in favour of opportunistic deposit-feeder ones. At greater depth, the organic matter and mud accumulation, the consequent reduced dissolved oxygen and Eh, in addition to the increase in seabird-mediated trace element concentration [25], lead to a total decline in benthic fauna, representing the final step in the biological succession in harsh environments and certainly producing a lower ecological status than in the other ponds, as the BITS index indicated.

## Conclusions

In the Marinello ponds we found evidence for bottom-up control of guanotrophication effects on soft bottom macrobenthic communities, in terms of abundances, trophic mode and environmental quality of the ponds. Macrobenthic patterns showed high variability between ponds at different distances from the gull colony and were significantly influenced by both trophic and geomorphological condition. The patterns observed allow us to state that the response of the soft-bottom benthic fauna to guanotrophication may be associated with the typical response to severe eutrophication in coastal areas. The response is magnified in the deeper sites where geomorphological features cause greater accumulation of mud and organic matter, which lead to even more impoverished benthic communities. An increase in density, species abundance and diversity emerged at moderate nutrient and organic loading; as seabird-mediated loading increases, the macrobenthic response shifts toward high abundances of only a few opportunistic deposit feeders, up to azoic conditions.
